# *In Vitro* and *In Situ* Characterization of Fish Thymic Nurse Cells

**DOI:** 10.1155/1996/14738

**Published:** 1996

**Authors:** Emilio Flaño, Francisco Álvarez, Pilar López-Fierro, Blanca E. Razquin, Alberto J. Villena, Agustín G. Zapata

**Affiliations:** 1 Departamento de Biología Celular y Anatomía Facultad de Biología Universidad de León León 24071 Spain; 2 Departamento de Biología Celular Facultad de Ciencias Biológicas Universidad Complutense de Madrid Madrid 28040 Spain

**Keywords:** Thymic epithelium, thymic nurse cells, trout thymus

## Abstract

We present an enzyme- and immuno-cytochemical, and ultrastructural characterization of trout thymic nurse cells (TNCs). Our data suggest that isolated trout thymic multicellular complexes are epithelial cells with acidic compartments that may be involved in the processing of antigens and in the generation of the MHC-II proteins that these cell express, and also that isolated TNCs are the *In Vitro* equivalent of the pale and intermediate electronlucent epithelial cells located in the inner zone of the trout thymus, constituting indirect evidence of the phylogenetical relationships of the inner zone of the teleost thymus with the thymic cortex of higher vertebrates.


*Developmental Immunology*, 1996, Vol 5, pp. 17–24
Reprints available directly from the publisher
Photocopying permitted by license only

© 1996 OPA (Overseas Publishers Association)
Amsterdam B.V. Published in The Netherlands
by Harwood Academic Publishers GmbH
Printed in Singapore

### *In Vitro* and *In Situ* Characterization of Fish Thymic Nurse Cells

EMILIO FLAÑO,[†]* FRANCISCO ÁLVAREZ,[†] PILAR LÓPEZ-FIERRO,[†] BLANCA E. RAZQUIN,[†] ALBERTO J. VILLENA,[†] and AGUSTÍN G. ZAPATA[‡]

[†]*Departamento de Biología Celular y Anatomía, Facultad de Biología, Universidad de León, 24071 León, Spain*

[‡]*Departamento de Biología Celular, Facultad de Ciencias Biológicas, Universidad Complutense de Madrid, 28040 Madrid, Spain*

We present an enzyme- and immuno-cytochemical, and ultrastructural characterization of trout thymic nurse cells (TNCs). Our data suggest that isolated trout thymic multicellular complexes are epithelial cells with acidic compartments that may be involved in the processing of antigens and in the generation of the MHC-II proteins that these cell express, and also that isolated TNCs are the *in vitro* equivalent of the pale and intermediate electron-lucent epithelial cells located in the inner zone of the trout thymus, constituting indirect evidence of the phylogenetical relationships of the inner zone of the teleost thymus with the thymic cortex of higher vertebrates.

KEYWORDS: Thymic epithelium, thymic nurse cells, trout thymus.

### INTRODUCTION

Multicellular complexes of thymocytes and stromal cells, including both epithelial cells and bone-marrow-derived cells, can be isolated *in vitro* from thymus. Several years ago, Wekerle et al. (1980) isolated *in vitro* from the mouse thymus thymocyte-epithelial-cell complexes, named thymic nurse cells (TNCs), in which the lymphocytes are enclosed within vacuoles lined by the epithelial-cell membrane. It is generally assumed that *in vitro* TNCs represent the *in vivo* association of epithelial cells with cortical thymocytes. Some authors have nevertheless reported lymphocyte-epithelial-cell complexes in lymphoid organs other than the thymus (Gerdes et al., 1983; Manconi et al., 1984; Wick and Oberhuber, 1986; Tsunoda and Kojima, 1987). The epithelial nature of TNCs was established by the presence of keratin bundles (Vakharia, 1983; de Waal Malefijt et al., 1986; Toussaint-Demylle et al., 1993), whereas thymocytes within TNCs are mainly immature double-positive $CD4^+$ $CD8^+$ cells (Ritter et al., 1981; Kyewski and Kaplan, 1982; Van Vliet et al., 1984; Kyewski et al., 1987), although some authors have reported more mature cells in the complexes (Vakharia, 1983; Hugo et al., 1988) and others also include double-negative $CD4^-$ $CD8^-$ cells (Singer et al., 1986; Kyewski, 1986; Wood et al., 1988).

Although TNCs have repeatedly been claimed to play a decisive role in T-cell maturation, their true functional significance is a matter of discussion. They have been involved in the establishment of positive (Farr and Anderson, 1985; Kyewski, 1986; Owen et al., 1986; Ron et al., 1986) and negative selection (Wick and Oberhuber, 1986; Lorenz and Allen, 1989; Speiser et al., 1992), although their formation is not dependent on TCR–MHC interaction (Boyd et al., 1993; Aguilar et al., 1994). Recently, Aguilar et al. (1994) have concluded their participation in the process of thymocyte apoptosis or in the clearing of apoptotic thymocytes.

TNCs have been found in the thymus of all higher vertebrates studied (Wick and Oberhuber, 1986; Penninger et al., 1990, 1994; Boyd et al., 1993), but direct evidence of their existence in ectotherms is lacking. In *Rana pipiens* tadpole thymi, Holtfreter and Cohen (1987) described thymocyte-stromal-cell complexes with regard only to their external appearance, size, and number of internalized thymocytes, including TNCs, and also thymic rosette cells, which contained macrophages and presumably dendriticlike cells. In the teleost thymus, morphological studies have emphasized the intimate association

*Corresponding author.

between the epithelial cells and thymocytes (Pulsford et al., 1991; Álvarez, 1993), whereas thymocyte-macrophage complexes have been reported in both elasmobranches (Pulsford et al., 1984; Navarro, 1987) and teleosts (Fänge and Pulsford, 1985).

In this paper, we report for the first time the *in vitro* isolation of TNCs from the adult trout thymus, analyzing their cytochemical and ultrastructural characteristics and their presumptive relationships with thymocyte-epithelial-cell complexes observed *in situ*.

### RESULTS

### Light Microscopy

Isolated lymphoid-epithelial-cell complexes appeared as large round cells with a regular outline containing 1 to 11 (average 3 to 4) round lymphocytic nuclei. The nuclei of the epithelial cells were irregular in shape, eccentric, and poorly stained (Fig. 1).

### Enzyme- and Immuno-Cytochemical Analysis

Enzyme-Cytochemical Analysis

The lymphoid-epithelial-cell complexes showed ACPH (Fig. 2), AKPH (Fig. 3), and ANAE (Fig. 4) enzyme activities. Controls for the enzymatic activities assayed were always negative.

Immuno-Cytochemical Analysis

The lymphoid-epithelial-cell complexes showed certain cross-reactivity with a mouse monoclonal antibody (myc-16$^+$) raised against chicken Ia antigens (Fig. 5) and were strongly positive for keratin (Fig. 6). Cell complexes incubated without primary antibodies were always negative.

### Transmission Electron Microscopy

Isolated Cells

Trout thymic lymphoid-epithelial-cell complexes

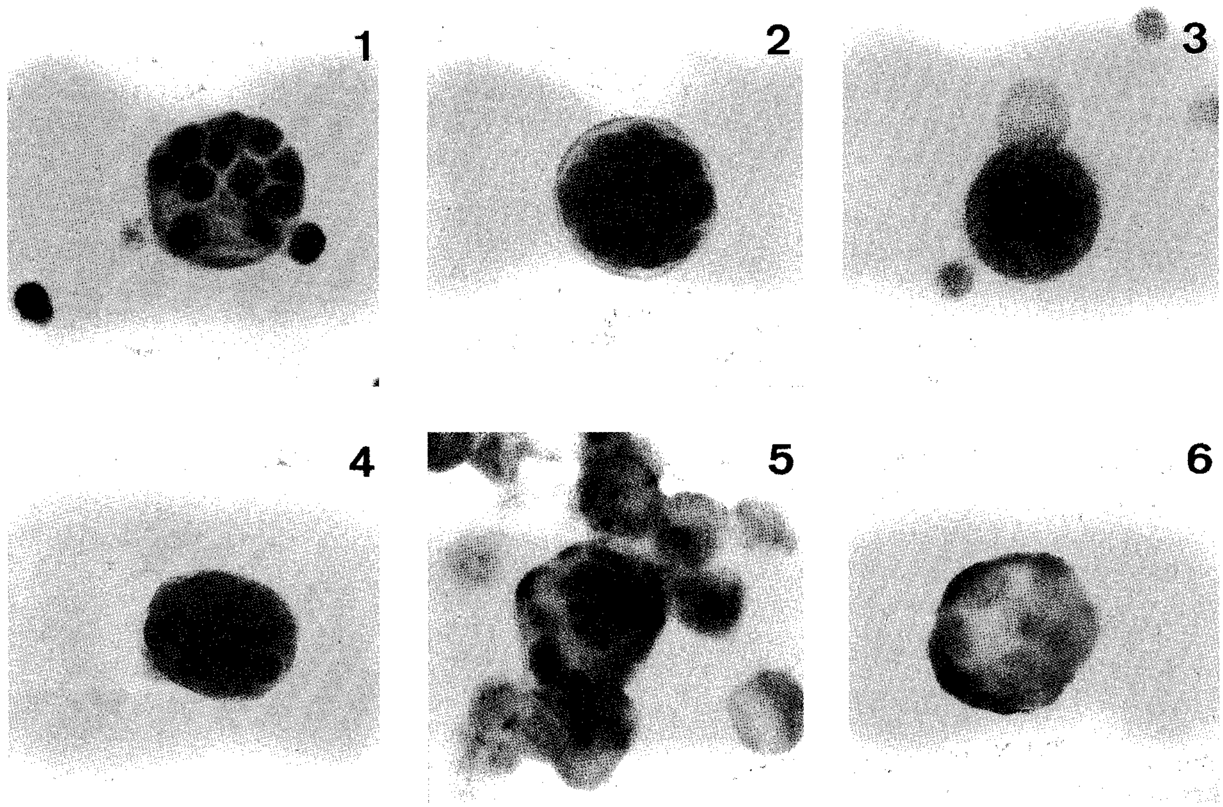

FIGURES 1 to 6 (1) TNC stained with haematoxilin-eosin (×1,150). (2) TNC stained for ACPH activity (×1,150). (3) TNC stained for AKPH activity (×1,150). (4) TNC stained for ANAE activity (×1,050). (5) TNC positive for the myc-16$^+$ monoclonal antibody (×1,150). (6) TNC positive for the pan-keratin polyclonal antibody (×1,150).

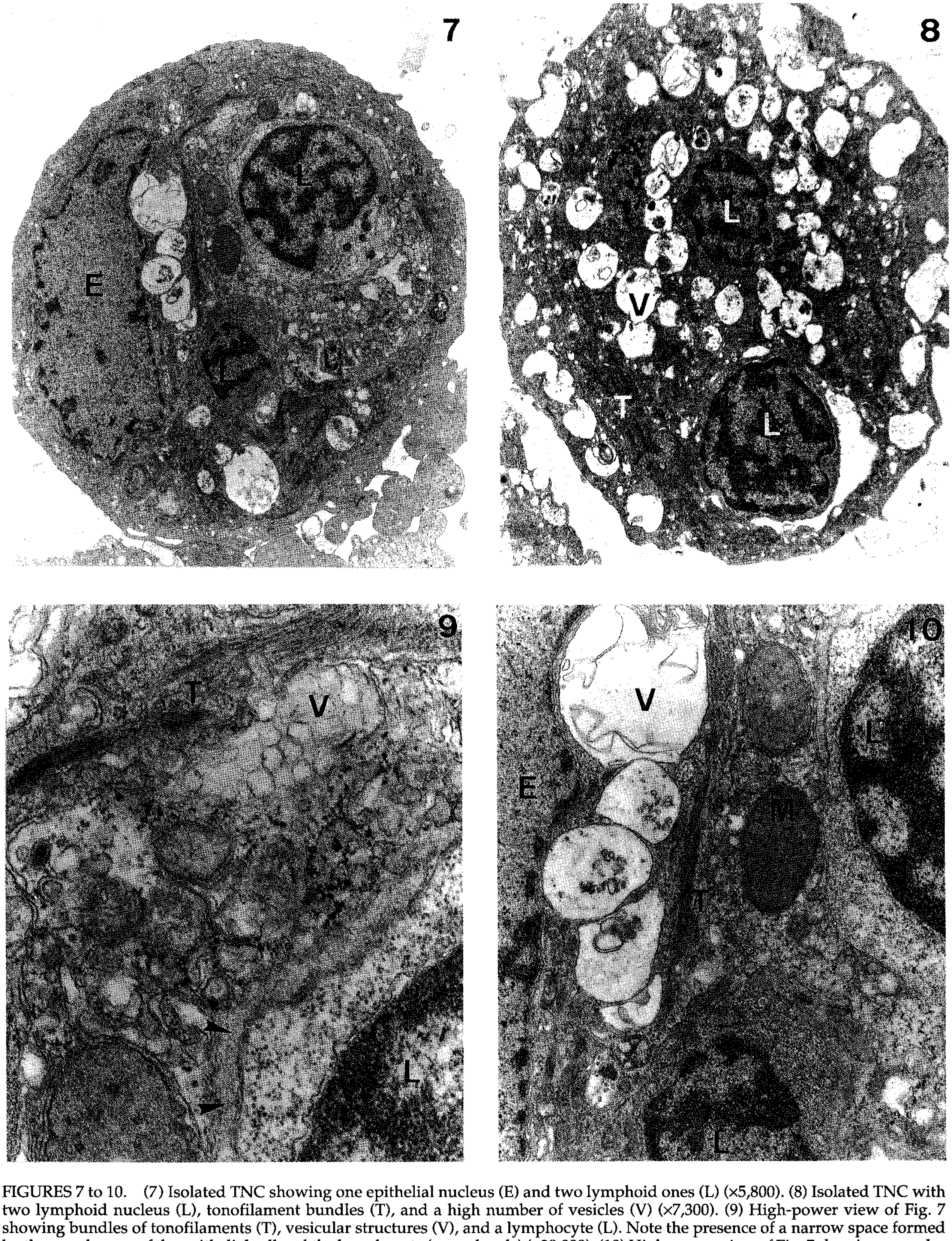

FIGURES 7 to 10. (7) Isolated TNC showing one epithelial nucleus (E) and two lymphoid ones (L) (×5,800). (8) Isolated TNC with two lymphoid nucleus (L), tonofilament bundles (T), and a high number of vesicles (V) (×7,300). (9) High-power view of Fig. 7 showing bundles of tonofilaments (T), vesicular structures (V), and a lymphocyte (L). Note the presence of a narrow space formed by the membranes of the epithelial cell and the lymphocyte (arrowheads) (×38,000). (10) High-power view of Fig. 7 showing vacuolar structures (V), bundles of tonofilaments (T), mitochondria (M), one epithelial nucleus (E), and two lymphoid ones (L) (×15,100).

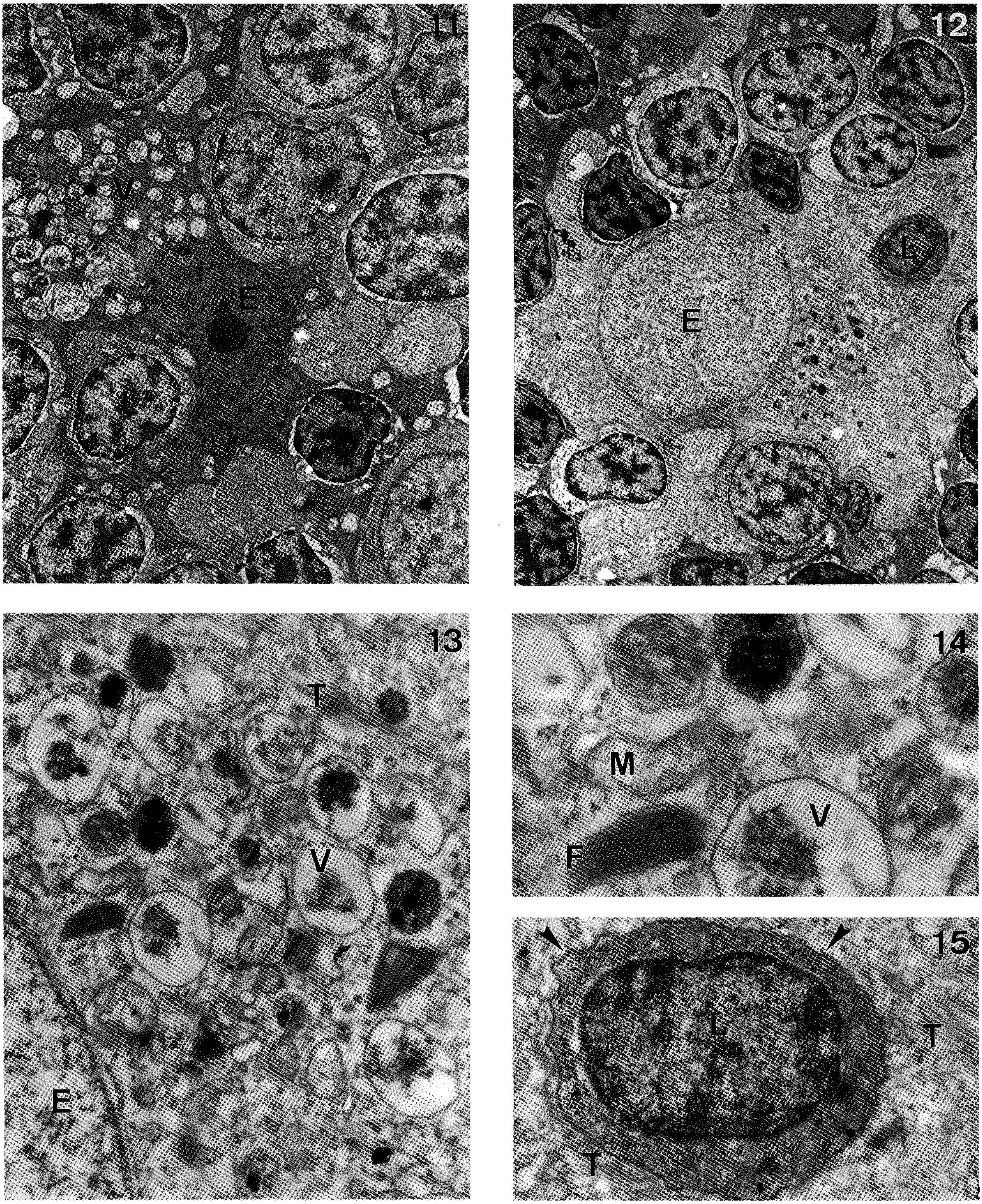

FIGURES 11 to 15. (11) Intermediate electron-lucent epithelial cell (E) of the thymic inner zone with an intracytoplasmic lymphocyte (L) and numerous vacuolar structures (V) (×4,100). (12) Pale epithelial cell (E) of the thymic inner zone with an intracytoplasmic lymphocyte (L) (×3,700). (13) High-power view of Fig. 12 showing the epithelial nucleus (E), numerous vesicular structures with a heterogeneous content (V), and tonofilament bundles (T) (×18,000). (14) Detail of Fig. 13 showing the vesicular structures (V), a vesicle with a fibrous content (F), and a mitochondria (M) (×39,300). (15) High-power view of Fig. 12 showing an intracytoplasmic lymphocyte (L) surrounded by a double membrane (arrowheads) and tonofilaments (T) (×16,000).

consisted of one epithelial cell that embraced several lymphoid cells. The epithelial cell showed a euchromatic or moderately heterochromatic nucleus ovoid or irregular in shape, sometimes with indentations (Figs 7 and 8). The cytoplasm contained numerous mitochondria, free ribosomes, cisternae of rough endoplasmic reticulum, and large tonofilament bundles that frequently surrounded the vacuoles containing thymocytes (Figs 9 and 10). In addition, the epithelial cell showed numerous large electron-lucent and electron-dense membranous vesicles resembling both lysosomes and endosomes (Figs 7 to 10). Some vacuoles with internal membranous structures were also present throughout the cytoplasm (Figs 9 and 10).

Within the TNCs, several thymocytes were individually enclosed in vacuoles lined by the epithelial-cell membrane. Membranes of both cell types were in close contact, forming a narrow intercellular space (Fig. 9). Intra-TNC thymocytes, without signs of apoptosis or degeneration, had round or avoid heterochromatic nuclei and homogeneous, electron-dense cytoplasm containing numerous free ribosomes and mitochondria (Figs 7 and 9).

### *In Situ* Lymphoid-Epithelial Cell Complexes

Previously, we had defined *in situ* seven different types of epithelial cells in the trout thymus according to their location in the organ, morphology, histochemical reactivity, and ontogenetical development (Castillo et al., 1990, 1991). Two of these epithelial cell types, pale and intermediate electron-lucent, which occupied the so-called inner zone of the thymus, appeared frequently in close association with thymocytes apparently enclosed in their cytoplasm (Figs 11 and 12). The intermediate electron-lucent epithelial cells had numerous cell processes extending between neighboring thymocytes and other epithelial cells (Fig. 11). Their cytoplasm contained microfilaments, elements of the Golgi apparatus, profiles of rough endoplasmic reticulum, a few electron-dense vesicles, as well as numerous vesicles with a heterogeneous electron-pale content (Fig. 11). The nucleus, irregular in shape, was moderately heterochromatic, with a prominent nucleolus. Engulfed thymocytes frequently appeared in the epithelial cytoplasm in close contact with pale vesicles (Fig. 11). On the other hand, pale epithelial cells had euchromatic nuclei and ovoid electron-lucent cytoplasm. They contained free ribosomes, mitochondria, some groups of dictyosomes, rough endoplasmic reticulum, and large vesicles with a heterogeneous content (Figs 12 to 15). In addition, bundles of tonofilaments, sometimes closely apposed to thymocyte-containing vacuoles (Fig. 15), and vesicles with a fibrous content, appeared in these cells (Fig. 14).

Intraepithelial thymocytes were individually enclosed in vacuoles lined by the epithelial-cell membrane (Fig. 15). They showed no signs of cell degeneration, containing a large number of free ribosomes and mitochondria.

### DISCUSSION

Our results demonstrate for the first time that the trout thymus contains thymocyte-epithelial cell complexes, which can be isolated *in vitro*, resembling morphologically the TNCs described in higher vertebrates. As previously reported in both mammals and birds (Wekerle et al., 1980; Vakharia, 1983; Penninger et al., 1994), the teleost TNCs consist of epithelial cells with numerous mitochondria, profiles of rough endoplasmic reticulum, large bundles of tonofilaments, and membranous organelles resembling endo- and lysosomes. In addition, they are keratin-positive and exhibit acid and alkaline phosphatase and nonspecific esterase activities. These morpho-cytochemical features suggest that trout TNCs have a high metabolic activity and contain presumably acidic cytoplasmic compartments, important for antigen processing, as recently demonstrated in chicken TNCs (Penninger et al., 1994).

On the other hand, our *in situ* electron microscopy study confirms previous results (Pulsford et al., 1991; Álvarez, 1993) concerning the occurrence of thymocyte-epithelial-cell complexes in the teleost thymus. They correspond to both pale and intermediate electron-lucent epithelial cells of the inner zone of trout thymus. Indeed, they resemble morphologically the so-called type 2 (pale) and type 3 (intermediate) epithelial cells of the human thymus (Wijngaert et al., 1984), which have been proposed as the TNCs found in suspensions (Van de Wijngaert et al., 1984; Boyd et al., 1993). Thus, because mammalian TNCs seem to correspond mainly to cortical epithelial cells (Ritter et al., 1981; Kyewski and Kaplan, 1982; Van Vliet et al., 1984; Nabarra and Adrianarison, 1987), although their relationship to subcapsular (Kyewski and Kaplan, 1982; Kaneshima et al., 1987; Mizutami et al., 1987) and medullar epithelium has also been mentioned (Hugo et al., 1988), our results indirectly demonstrate the phylogenetical relationships of the inner zone of the teleost thymus with the thymic cortex of higher vertebrates, a fact repeatedly suggested by our group (Zapata, 1981; Castillo et al., 1990, 1991). It is, however, difficult, from a merely morphological study, to establish correlations between the *in vitro* isolated TNCs and these lymphocyte-epithelial-cell clusters identified *in situ*. Moreover, some authors have mentioned a certain heterogeneity in enriched TNCs from the human thymus (Ritter et al., 1981).

All phenotypical studies remark on the expression of class I and class II MHC on higher-vertebrate TNCs (Wekerle et al., 1980; Ritter et al., 1981; Boyd et al., 1984), although the use of trypsine treatment during the isolation of cell clusters modifies this expression (Kyewski and Kaplan, 1982). Unfortunately, there are no available reagents to detect MHC molecules in teleost fish, although recent molecular data demonstrated the existence of both class I and class II antigens in them (Kromenberg et al., 1994). By using cross-reactive monoclonal antibodies, Kaufman et al. (1990) detected MHC-like molecules in some nonmammalian vertebrates. On this same basis, we have demonstrated a slight expression of MHC class II antigens in trout TNCs using an anti-chicken class II MHC molecule monoclonal antibody.

The expression of class II molecules and the existence of an acidic cytoplasmic compartment, as indicated by our enzyme-histochemical and ultrastructural results, suggest some capacity of teleost TNCs for antigenic processing and presentation, as recently reported for chicken TNCs (Penninger et al., 1994). Nevertheless, the TCR–MHC interactions are apparently not necessary for the formation of mouse TNCs (Boyd et al., 1993) and, although most authors emphasize a role for TNCs in intra-thymic T-cell maturation, their functional significance is really obscure.

### MATERIALS AND METHODS

### Animals

Thymi from rainbow trout, *Oncorhynchus mykiss*, and brown trout, *Salmo trutta*, were used for cell isolation and histological examination. Fish were aged by scalimetry and were between 1 and 3 years. No differences were seen between the two species studied in any of the experimental systems used. Fish were anesthesized with MS-222 (Sandoz Pharma Ltd., Bâle, Switzerland), blood was extracted from the caudal sinus, and the thymi were dissected.

### Cell Isolation

The thymi were mechanically minced in ice-cold phosphate-buffered saline (PBS), and the supernatant containing free cells was discarded. The remaining tissue fragments were incubated in PBS for 10 min at 15°C with stirring, and the supernatant discarded again. After this mechanical dissociation, the tissue fragments were digested with 0.6 mg/ml type Ia collagenase (Sigma, St. Louis) in PBS for 15 min at 15°C under agitation. The supernatant was discarded and this step was repeated three times, the supernatants of the two last steps being harvested. After collagenase digestion, the remaining tissue fragments were trypsinized (1% trypsin-EDTA in PBS; Boehringer-Mannheim) for 30 min at 15°C under gentle agitation. Collection of TNC was achieved using a modification of the method of Wekerle et al. (1980). TNC-containing fractions were enriched for TNC by one 1-g sedimentation over FCS at 4°C for 15 min. After sedimentation, the top layer was discarded, and the rest of the suspension was centrifuged at 250 g for 10 min at 4°C and resuspended in PBS.

### Routine Histology

The isolated cells were placed in drops onto slides, air dried, and stained with haematoxylin-eosin for routine examination and identification of multicellular complexes.

### Enzyme- and Immuno-Cytochemical Analysis

The assayed enzymatic activities and the substrates used are summarized in Table 1. Negative controls for the specificity of the enzymatic reactions were established using incubation media in which the corresponding substrates were lacking.

For immunodetection, cells were fixed in acetone for 10 min at room temperature, air dried, and processed by the indirect immunoperoxidase technique using 3,3-diaminobenzidine-tetrahydrochloride (Sigma) as a coupling reagent. Controls were systematically performed by omission of the first antibody, and endogenous peroxidase activity was previously inhibited by incubation in 0.3% hydrogen peroxide in methanol for 10 min at room temperature. Keratins were identified using a pan-keratin rabbit polyclonal antibody (Dako, Denmark), and MHC class II proteins were detected using cross-reactivity of a mouse monoclonal antibody myc-16$^+$ raised against chicken Ia antigens (kindly provided by Dr M. Cooper). As secondary antibodies, a goat anti-rabbit Ig (Sigma) and a rabbit anti-mouse IgG antiserum (Dako), peroxidase-conjugated, were used, respectively. Before their use on isolated cells, all antibodies were checked for their specificity *in vivo* on acetone-fixed cryosections of trout thymus.

### Transmission Electron Microscopy

Isolated cells and thymi were fixed in 2% glutaraldehyde in 0.1 M cacodylate buffer, pH 7.2, postfixed with 1% osmium tetroxide in the same buffer, dehydrated in acetone series, counterstained with 1% uranyl acetate in 70% acetone, and embedded in Araldite (Durcupan, ACM, Fluka, Switzerland). One-micrometer thick sections were stained with an aqueous solution of toluidine blue in borax to select the most suitable areas. Ultrathin sections were obtained with a Reichert-Jung UM-3 ultratome, counterstained with lead citrate, and examined in a JEOL-EM1010 electron microscope at 60 kV.

### ACKNOWLEDGMENTS

The authors thank Dr M. Cooper for kindly providing the myc-16$^+$ monoclonal antibody. E. Flaño and F. Álvarez are supported by EC contract AIR1 CT92 0036.

*(Received September 6, 1995)*

*(Accepted January 3, 1996)*

### REFERENCES

Aguilar L.K., Aguilar-Cordova E., Cartwright J. Jr., and Belmont J.W. (1994). Thymic nurse cells are sites of thymocyte apoptosis. J. Immunol. **152**: 2645–2651.

Álvarez F. (1993). Immunobiología de salmónidos. Análisis morfológico de los mecanismos de defensa de *Salmo trutta* contra la infección por *Saprolegnia* sp. Estudio del tegumento y los órganos linfoides. Doctoral thesis, University of León, León, Spain.

Barka T., and Anderson P.J. (1962). Histochemical methods for acid phosphatase using hexazonium pararosanilin as coupler. J. Histochem. Cytochem. **10**: 741–753.

Boyd R.L., Oberhuber G., Hala K., and Wick G. (1984). Obese strain (os) chickens with spontaneous autoimmune tyroiditis have a deficiency in thymic nurse cells. J. Immunol. **132**: 718–724.

Boyd R.L., Tucek C.L., Godfrey D.I., et al. (1993). The thymic microenvironment. Immunol. Today **14**: 445–459.

Burstone M.S.(1958). Histochemical comparison of naphthol AS-phosphates for the demonstration of phosphatases. J. Natl Cancer Inst. **20**: 601–615.

Castillo A., López-Fierro P., Zapata A., Villena A., and Razquin B. (1991). Post-hatching development of the thymic epithelial cells in the rainbow trout *Salmo gairdneri*: An ultrastructural study. Amer. J. Anat. **190**: 299–307.

Castillo A., Razquin B.E., López-Fierro P., Álvarez F., Zapata A., and Villena A. (1990). Enzyme- and immuno-histochemical study of the thymic stroma in the rainbow trout, *Salmo gairdneri* Rich. Thymus **15**: 153–166.

De Waal Malefijt R., Leene W., Rohall P.J.M., Wormmeester J., and Hoeben K.A. (1986). T cell differentiation within thymic nurse cells. Lab. Invest. **55**: 25–34.

Fänge R. and Pulsford A. (1985). The thymus of the angler fish *Lophius piscatorius*: An ultrastructural study. *In*: Fish immunology, Manning M.J., and Tatner M.F., Eds. (London: Academic Press), pp. 293–311.

Farr A.G., and Anderson S.K. (1985). Epithelial cell heterogeneity in the murine thymus: Fucose-specific lectins bind medullary epithelial cells. J. Immunol. **134**: 2971–2977.

Gerdes J., Stein H., Masm D.Y., and Ziegler A. (1983). Human dendritic reticulum cells of lymphoid follicles: Their antigenic profile and their identification as multinucleated giant cells. Virchows Arch. (Cell. Pathol.) **42**: 161–172.

Holtfreter H.B., and Cohen N. (1987). *In vitro* behavior of thymic nurse cell-like complexes from mechanically and enzymatically dissociated frog tadpole thymus. Amer. J. Anat. **179**: 342–355.

Hugo P., St-Pierre Y., and Potworowsky E.F. (1988). Characterization of the *in vitro* interaction between thymocytes and a medullary thymic epithelial cell line. Thymus **12**: 27–37.

Kaneshima H., Ito M., Asai J., Taguchi O., and Hiai H. (1987). Thymic epithelial cell subpopulations in mice defined by monoclonal antibodies. Lab. Invest. **56**: 372–380.

Kaufman J., Ferrone S., Flajnik M., Kilb M., Völk H., and Parisot R. (1990). MHC-like molecules in some non-mammalian vertebrates can be detected by some cross-reactive monoclonal antibodies. J. Immunol. **144**: 2273–2280.

Kronenberg M., Brines R., and Kaufman J. (1994). MHC evolution: A long term investment in defense. Immunol. Today **4**: 4–6.

TABLE 1

| Enzymatic activity[a] | Substrate | Dose(mg/ml) | Incubation medium | Reference |
|---|---|---|---|---|
| ACPH | Naphthol *AS-BI* phosphate | 0.5 | pH 5 | Barka and Anderson, 1962 |
| AKPH | Naphthol *AS-BI* phosphate | 0.1 | pH 8 | Burstone, 1958 |
| ANAE | α-naphthyl acetate | 0.25 | pH 6.5 | Pearse, 1972 |

[a]ACPH: acid phosphatase. AKPH: alkaline phosphatase. ANAE: nonspecific a-naphthyl acetate esterase.

Kyewski B.A., and Kaplan H.S. (1982). Lymphoepithelial interations in the mouse thymus: Phenotypic and kinetic studies on thymic nurse cells. J. Immunol. **128**: 2287–2294.

Kyewski B.A. (1986). Thymic nurse cells: Possible sites of T-cell selection. Immunol. Today 7: 374–379.

Kyewski B.A., Momburg F., and Schirrmacher V. (1987). Phenotype of stromal cell-associated thymocytes *in situ* is compatible with selection of the T cell repertoire at an "inmature" stage of thymic T cell differentiation. Eur. J. Immunol. **17**: 961–967.

Lorenz R.G., and Allen P.M. (1989). Thymic cortical epithelial cells can present self antigens *in vivo*. Nature **337**: 560–562.

Manconi P.E., Ennas M.G., Murrn M.R. Caddedu G., Tore G., and Lantini M.S. (1984). Epithelial-like cells containing lymphocytes (nurse cells) in human adenoids and tonsils. Thymus **6**: 351–357.

Mizutami S., Watt S.M., Robertson D., Hussein S., Healy L.E., Furley A.J.V., and Greaves M.F. (1987). Cloning of human thymic subcapsular-cortex epithelial cells with T-lymphocyte binding sites and hemopoietic growth factor acticity. *Proc. Natl Acad. Sci. USA* **84**: 4999–5003.

Nabarra B., and Adrianarison I. (1987). Ultrastructural studies of thymic reticulum. I. Epithelial component. Thymus **9**: 95–121.

Navarro R. (1987). Ontogenia de los órganos linfoides de *Scyliorhinus canicula*. Estudio ultraestructural. Master thesis, Complutense University, Madrid.

Owen J.J.T., Jenkison E.J., and Kingston R. (1986). Thymic stem cells: Their interaction with the thymic stroma and tolerance induction. Curr. Topics Microbiol. Immunol. **126**: 35–41.

Pearse A.G.E. (1972). Histochemistry. Theoretical and applied (Edinburg: Churchill Livingstone).

Penninger J., Hala K., and Wick G. (1990). Intrathymic nurse cell lymphocyte can induce a specific graft-versus-host reaction. J. Exp. Med. **172**: 521–529.

Penninger J., Rieker T., Romani N., et al. (1994). Ultrastructural analysis of thymic nurse cell epithelium. Eur. J. Immunol. **24**: 222–228.

Pulsford A., Fänge R., and Zapata A. (1991). The thymic microenvironment of the common sole, *Solea solea*. Acta Zool. (Stockholm) **72**: 209–216.

Pulsford A., Morrow W.J.W., and Fánge R. (1984). Ultrastructural studies on the thymus of the dogfish, *Scyliorhinus canicula* L.J. Fish Biol. **25**: 353–360.

Ritter M.A., Sauvage C.A., and Cotmore S.F. (1981). The human thymus microenvironment: *In vivo* identification of thymic nurse cells and other antigenically-distinct subpopulations of epithelial cells. Immunology **44**: 439–446.

Ron Y., Lo D., and Spent J. (1986). T cell specificity in twice-irradiated F1-into-parent bone marrow chimeras: Failure to detect a role for immigrant marrow-derived cells in imprinting intrathymic H-2 restriction. J. Immunol. **137**: 1764–1771.

Singer K.H., Wolf L.S., Lobach D.F., Denning S.M., Tuck D.T., Robertso A.L., and Haynes B.F. (1986). Human thymocytes bind to autologus and allogenic thymic epithelial cells *in vitro*. *Proc. Natl Acad. Sci. USA* **83**: 6588-6592.

Speiser D.E., Pircher H., Ohashi P.S., Kyburz D., Hengartner H., and Zinkernagel R.M. (1992). Clonal deletion induced by either radioresistant thymic host cells or lymphohemopoietic donor cells at different stages of class I-restricted T-cell ontogeny. J. Exp. Med. **175**: 1277–1284.

Toussaint-Demylle D., Scheiff J.M., and Haumont S. (1993). Thymic nurse cells in culture: Morphological and antigenic characterization. Cell Tis. Res. **272**: 343–354.

Tsunoda R., and Kojima M. (1987). A light microscopical study of isolated follicular dendritic cell-clusters in human tonsils. Acta Pathol. Jpn **37**: 575–586.

Vakharia D.D. (1983). Demonstration of keratin filaments in thymic nurse cells and alloreactivity of TNC-T cell population. Thymus **5**: 43–52.

Van de Wijngaert F.P., Kendall M.D., Schuurman H.J., Rademakers L.H.P.M., and Kater L. (1984). Heterogeneity of epithelial cells in the human thymus. An ultrastructural study. Cell Tissue Res. **237**: 227–237.

Van Vliet E., Melis M., and Van Ewijk W. (1984). Immunohistology of thymic nurse cells. Cell. Immunol. **87**: 101–109.

Wekerle H., Ketelsen U.P., and Ernst M. (1980). Thymic nurse cells. Lymphoepithelial cell complexes in murine thymuses: Morphological and Serological characterization. J. Exp. Med. **151**: 925–944.

Wick G., and Oberhuber G. (1986). Thymic nurse cells: A school for alloreactive and autoreactive cortical thymocytes? Eur. J. Immunol. **16**: 855–858.

Wood G.W., Mauser L., Fleming R.H., Greenwood J., and Sandford T.H. (1988). J. Immunol. **141**: 2221–2229.

Zapata A. (1981). Lymphoid organs of teleost fish. I. Ultrastructure of the thymus of *Rutilus rutilus*. Dev. Comp. Immunol. **5**: 427–436.

